# Malnutrition, Overweight, and Obesity among Urban and Rural Children in North of West Azerbijan, Iran

**DOI:** 10.1155/2014/541213

**Published:** 2014-05-25

**Authors:** Sakineh Nouri Saeidlou, Fariba Babaei, Parvin Ayremlou

**Affiliations:** ^1^Food and Beverages Safety Research Center, Urmia University of Medical Science, Urmia, Iran; ^2^Urmia University of Medical Science, Urmia, Iran

## Abstract

*Introduction.* Malnutrition is one of the most important causes for improper physical and mental development of children. Childhood obesity is a worldwide public health problem. The increasing prevalence of childhood obesity has become a growing matter of public health concern worldwide. The aim of the current study was to determine the prevalence of malnutrition and obesity in children under 5 years old in Salmas district. *Methods.* The current study is a cross-sectional study conducted on 902 of children under 5 years old to assess the nutritional status in Salmas district and performed from 16 until 30 October, 2011, with the cooperation of the Office of Community Nutrition Improvement and the United Nations Children's Fund. ENA (Emergency Nutrition Assessment) and Spss software were used for data analysis. *Results.* 49.6% of children were boys and 50.4% were girls. The prevalence of malnutrition based on underweight, stunting, and wasting was estimated to be 2.3%, 7.3%, and 1.4% among children, respectively. Stunting was more common in rural areas and this difference was significant (*P* < 0.001). *Conclusion.* In this area stunting, overweight and obesity were the most important priorities that health officials must pay more attention to. ENA software has a special ability to determine the samples and clusters and is a simple, rapid, and accurate method, especially in epidemiological studies in the country, and can be a convenient tool and its use is suggested for the same studies.

## 1. Introduction

Malnutrition is one of the most important causes for improper physical and mental development of children [1]. One in every five children in the developing world is malnourished, and poor nutrition is associated with half of all child deaths worldwide [2]. Malnutrition in children causes an increase in morbidity and mortality and has an adverse effect on intellectual ability [3]. Globally, acute malnutrition causes more than 50% of childhood mortality in children under 5 years old, which implies that about 3.5 million children die of malnutrition each year [4].

**Figure 1:**
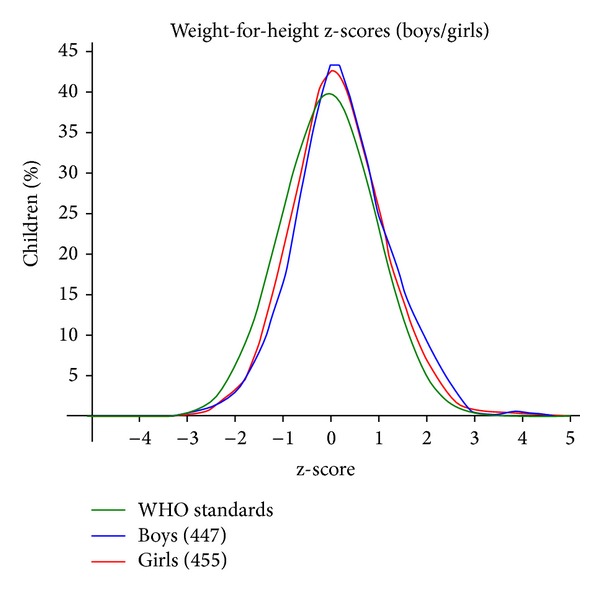
The graph of the normal distribution of weight for height in studied children in Salmas (mean ± SD = 0.21 ± 0.94).

**Figure 2:**
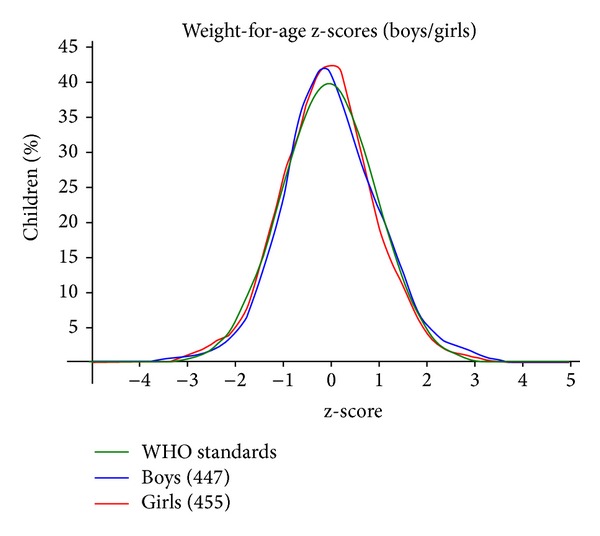
The graph of the normal distribution of weight for age in studied children in Salmas (mean ± SD = −0.01 ± 0.96).

Malnutrition measures in many ways. Clinical grading standard, weight-for-height (WFH) index (Figure 1), height-for-age (HFA) index, weight-for-age (WFA) index (Figure 2), body mass index, and skin fold thickness are to be used more frequently in the field [5]. In April 2006, the WHO released new global growth charts for infants and children as old as 5 years to replace the existing CDC/WHO international growth charts, which were based on the 1977 NCHS growth charts [6].

The worldwide malnutrition estimation rates indicate that 35.8% of preschool children in developing countries are underweight, 42.7% are stunted, and 9.2% are wasted [7]. Childhood obesity is a worldwide public health problem [8]. The increasing prevalence of childhood obesity has become a growing matter of public health concern worldwide. Obesity has increased from 4.2%, in 1990, to 6.7%, in 2010, worldwide and is expected to reach 9.1%, in 2020 [9, 10].

In Iran, like many of the other developing countries, the prevalence of obesity in children has been moving on [11]. According to a survey in West Azerbaijan, 8.7%, 7.5%, and 4.3% of the children aged less than five years suffered from stunting, wasting, and underweight, respectively [12]. The present study aimed at assessing the prevalence of malnutrition (underweight, stunting, wasting, overweight, and obesity) in under-five-year-old children in Salmas district.

## 2. Methods

The current study is a cross-sectional study which was conducted for assessing the nutritional status of children under 5 years old in Salmas district on the basis of national guide and has been performed from 16 until 30 October, 2011, with the cooperation of the Office of Community Nutrition Improvement and the United Nations Children's Fund (UNICEF). Using cluster sampling, the statistical population included 0–59-month-old children residing in the cities and villages of Salmas and by ENA software sample size was calculated with 5% confidence interval; 902 children were determined. In this study, children being mentally and physically retarded and having problems in terms of anthropometry were removed from the study and replaced by other children. The Institutional Review Board approved this study. For medical ethics, the parental consent form must be completed to conduct completed design. In this study, cluster sampling is used for selecting the samples. For this purpose, first, the total number of households residing in rural and urban areas and the total number of children between 0–59 months in Salmas city were cumulatively calculated. Then, in the ENA software items, in the planning part, the desired items were entered into sample size calculation as follows:

 the number of children under 5 years old: 19824 people,
 estimate of the prevalence number of malnutrition: 8.7,
 widespread confidence interval: 2.3,
 design effect: 1.5.

After entering the above information, 841 were determined as the sample size and, with 5% confidence interval for a sample sufficiency, the number of children was calculated as 885 children and, finally, in the study, 902 questionnaires were completed. Given that it was supposed to study 18 children in each cluster, 50 clusters were determined for this study. Then, the ratio of urban and rural populations in Salmas was calculated in this region; the urban population was 49% and the rural population was 51%. Therefore, 26 rural clusters and 24 urban clusters were determined and in the next stage the names of all villages and urban blocks were separately entered into the part of selecting clusters in ENA software according to the number of clusters. The required data were collected through measuring the height and weight and arm circumference of the children in the study, completing the questionnaire and interviews with mothers or caregivers of children. The scale used in this study was a single pan balance with the maximum capacity of 150 kg and accuracy of 100 gr. If possible, the child was directly weighed. If the baby was too small or cried so hard, first, the mother was weighed alone and then hugged the child. The scale automatically calculated the weight of the child by subtracting. Also, every day before starting work, to ensure the accuracy of the scale, the scale was tested using the control scale. The height measuring board was also used to measure height. The height of less-than-two-year children was measured in a supine and larger children were measured in standing position with an accuracy of one tenth of millimeter. The height measuring board was used for both positions. The middle of the left arm circumference in children from 6 to 59 months was measured using a special band of measuring arm circumference based on the following steps and was recorded in millimeters. Chi-square test was to be used for relationship independent variables (sex and region) with malnutrition.

## 3. Results

This study was done on 902 children under 5 years old including 49.6% being boys and 50.4% being girls. Most children were in the age group of 18–29 months (24.7%) and the lowest number was in the age group of 54–59 months (8.8%) (Table 1). Totally, the prevalence of malnutrition based on underweight, stunting, and wasting was estimated to be 2.3%, 7.3%, and 1.4% among children, respectively.

**Figure 3:**
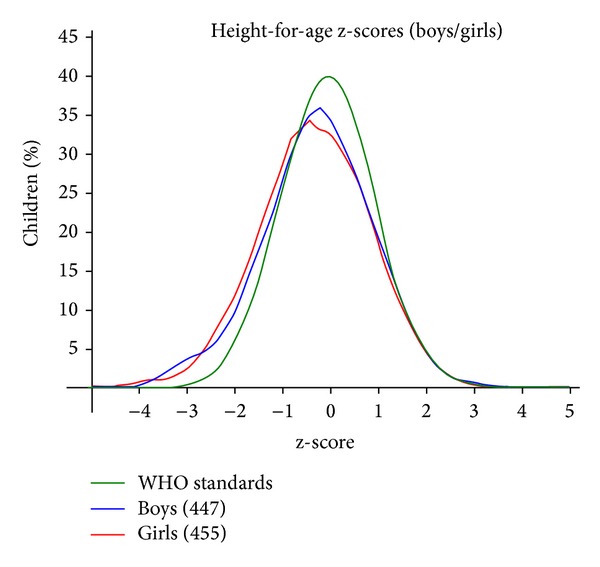
The graph of the normal distribution of height for age in studied children in Salmas (mean ± SD = −0.28 ± 1.08).

The results of the study showed that underweight in girls and rural areas was more common. Malnutrition under height for age in girls and boys in rural areas was more than that of the urban areas; also, we found that wasting index was not different in both sex and area. Stunting was more common than the other two malnutrition indexes (Table 2). Current study showed that prevalence of overweight and obesity in girls and rural areas was less than in boys and urban areas but this difference was not significant (Table 3). The relationship between gender and region with malnutrition showed that there was no statistical difference between sex and underweight, wasting, and stunting, but stunting was more common in rural areas and this different was significant (*P* < 0.001) (Table 4). The graph of the normal distribution of height for age in studied children in Salmas shows that distribution of height for age was skewed to left comparing with WHO standard (Figure 3).

## 4. Discussion

The aim of the current study was to assess the nutritional status of under-five-year-old children in urban and rural areas in Salmas district. The prevalence of malnutrition based on underweight, stunting, and wasting was estimated to be 2.3%, 7.3%, and 1.4% among children, respectively, in Salmas district. In a study in West Azerbaijan province by Farrokh-Eslamlou, prevalence of underweight, stunting, and wasting was estimated to be 4.3%, 8.7%, and 7.5%, respectively [12]. In another study, by Veghari, malnutrition was observed in 3.20%, 4.93%, and 5.13% based on underweight, stunting, and wasting, respectively [13]. In another study in Khorasan province Northeast of Iran the rate of underweight, stunting, and wasting was reported to be 7.5%, 12.5%, and 4.4%, respectively [7]. According to the UNICEF report, 11%, 15%, and 5% of under-five-year-old Iranian children suffer from underweight, stunting, and wasting up, respectively [13]. Results of current study showed that underweight in girls and rural areas was common more which is consistent with other studies [7, 13]. Malnutrition based on height for age in both girls and boys in rural areas was more than urban areas which is due to the poor economic status, cultural status, income level, food behavior, and less health care in rural areas that are known as the risk factor for malnutrition. Our findings show that there was no statistical difference between sex and underweight, wasting, and stunting, but we found statistically significant differences between stunting and region where stunting was more common in rural areas which is consistent with previous studies in Iran [12] and stunting is still highly prevalent in underdeveloped and developing countries [14].

In current study, prevalence of obesity and overweight in children was 1.3% and 5.1%, respectively. The prevalence of overweight and obesity is increasing worldwide and has become a public health challenge [10, 15]. The tracking of childhood overweight and associated health consequences into adulthood is of concern; several serious physical conditions are associated with overweight, especially obesity, among children including asthma, sleep problems, cardiovascular diseases, and type 2 diabetes. Prevalence of obesity and overweight in boys and urban areas was more than girls and rural areas but this difference was not significant.

## 5. Conclusion

In this area, stunting, overweight, and obesity are the most important priorities that health officials must pay more attention to. Given the differences between various provinces and regions of the country which are a result of the differences between the levels of development in these areas, the necessity of designing and implementing targeted strategies are required for different areas. It is worth noting that the present study has been conducted in a single period and in only one city of each province and using ENA software; therefore, the judgment about the whole province requires general investigation in all cites of the province and the obtained results are solely applied to these three cities. This study, also, showed that the ENA software has a special ability to determine the samples and clusters and is a simple, rapid, and accurate method, especially in epidemiological studies compared to other methods that were used in studies in our country which can be a convenient tool and its use is suggested for the same studies. Also, the quality control of the performed activities by the teams in the field is another distinctive feature of this software which is considered of high importance and emphasizes the use of this software.

## Figures and Tables

**Table 1:** Characterize of children 6–59 months in Salmas 2011.

Age (month)	Boy (%)	Girl (%)	Total (%)
6–17 months	78 (45.6)	93 (54.4)	171 (19)
18–29 months	109 (48.9)	114 (51.1)	223 (24.7)
30–41 months	114 (53.3)	100 (46.7)	214 (23.7)
42–53 months	105 (48.8)	110 (51.2)	215 (23.8)
54–59 months	41 (51.9)	38 (48.1)	79 (8.8)

Total	447 (49.6)	455 (50.4)	902 (100)

**Table 2:** The distribution of nutritional status (underweight, stunting, and wasting) in children in Salmas.

Index	Area				
	Urban		Rural		Total
	Boy *N* = 225 (%)	Girl *N* = 207 (%)	Boy *N* = 222 (%)	Girl *N* = 248 (%)	*N* = 902 (%)
Underweight (<−2 *z*-score)	5 (2.2)	2 (1)	3 (1.4)	11 (4.4)	21 (9)
Moderate underweight (<−2 *z*-score and ≥−3 *z*-score)	4 (1.8)	2 (1)	2 (0.9)	11 (4.4)	19 (8.1)
Severe underweight (<−3 *z*-score)	1 (0.4)	0 (0)	1 (0.5)	0 (0)	2 (0.9)
Stunting (<−2 *z*-score)	9 (4)	8 (3.9)	22 (9.9)	27 (10.9)	66 (28.7)
Moderate stunting (<−2 *z*-score and ≥−3 *z*-score)	5 (2.2)	7 (3.4)	19 (8.6)	22 (8.9)	53 (23.1)
Severe stunting (<−3 *z*-score)	2 (1.8)	1 (0.5)	1 (1.4)	5 (2)	9 (5.7)
Wasting (<−2 *z*-score)	4 (1.8)	3 (1.4)	3 (1.4)	3 (1.2)	13 (5.8)
Moderate wasting (<−2 *z*-score and ≥−3 *z*-score)	4 (1.8)	3 (1.4)	2 (0.9)	2 (0.8)	11 (4.9)
Sever wasting (<−3 *z*-score)	0 (0)	0 (0)	1 (0.5)	1 (0.4)	2 (0.9)

**Table 3:** The distribution of BMI in sex and area.

BMI	Gender		Area	
	Boy *N* = 447 (%)	Girl *N* = 455 (%)	Urban *N* = 432 (%)	Rural *N* = 470 (%)
Normal (<2 *z*-score)	418 (93.5)	438 (96.2)	402 (93.1)	454 (96.7)
Overweight (≥2 *z*-score and ≤3 *z*-score)	22 (4.9)	14 (3.1)	23 (5.3)	13 (2.7)
Obesity (≥3 *z*-score)	7 (1.6)	3 (0.7)	7 (1.6)	3 (0.6)

*P* value*	0.3		0.2	

*Chi-square test.

**Table 4:** Comparison of status and proportion of malnourished children by gender and region in Salmas.

Variable	Weight for age		Height for age		Weight for height	
	Normal (%)	Malnourished (%)	Normal (%)	Malnourished (%)	Normal (%)	Malnourished (%)
Gender						
Girl	442 (97.1)	13 (2.9)	420 (92.3)	35 (7.7)	449 (98.7)	6 (1.3)
Boy	439 (98.20)	8 (1.8)	416 (93.1)	31 (6.9)	440 (98.4)	7 (1.6)
*P* value	**0.29**		**0.66**		**0.75**	
Area						
Urban	426 (98.4)	7 (1.6)	416 (96.1)	17 (3.9)	426 (98.4)	7 (1.6)
Rural	455 (97)	14 (3)	429 (89.6)	49 (10.4)	463 (98.7)	6 (1.3)
*P* value*	**0.17**		**<0.001**		**0.67**	

*Chi-square test.

## References

[1] Das S, Rahman RM (2011). Application of ordinal logistic regression analysis in determining risk factors of child malnutrition in Bangladesh. *Nutrition Journal*.

[2] Psaki S, Bhutta ZA, Ahmed T (2012). Household food access and child malnutrition: results from the eight-country MAL-ED study. *Population Health Metrics*.

[3] Sharghi A, Kamran A, Faridan M (2011). Evaluating risk factors for protein-energy malnutrition in children under the age of six years: a case-control study from Iran. *International Journal of General Medicine*.

[4] Park S-E, Kim S, Ouma C, Loha M, Wierzba TF, Beck NS (2012). Community management of acute malnutrition in the developing world. *Pediatric Gastroenterology, Hepatology & Nutrition*.

[5] de Onis M, Onyango AW, Borghi E, Garza C, Yang H (2006). Comparison of the World Health Organization (WHO) child growth standards and the national center for health statistics/WHO international growth reference: implications for child health programmes. *Public Health Nutrition*.

[6] Mei Z, Ogden CL, Flegal KM, Grummer-Strawn LM (2008). Comparison of the prevalence of shortness, underweight, and overweight among US children aged 0 to 59 months by using the CDC 2000 and the WHO 2006 growth charts. *Journal of Pediatrics*.

[7] Payandeh A, Saki A, Safarian M, Tabesh H, Siadat Z (2013). Prevalence of malnutrition among preschool children in northeast of Iran, a result of a population based study. *Global Journal of Health Science*.

[8] Martínez-Vizcaíno V, Martínez MS, Pacheco BN (2012). Trends in excess of weight, underweight and adiposity among Spanish children from 2004 to 2010: the Cuenca study. *Public Health Nutrition*.

[9] Muhihi A (2013). Prevalence and determinants of obesity among primary school children in Dar es Salaam, Tanzania. *Public Health*.

[10] van Grieken A, Renders CM, Wijtzes AI, Hirasing RA, Raat H (2013). Overweight, obesity and underweight is associated with adverse psychosocial and physical health outcomes among 7-year-old children: the “be active, eat right” study. *PLoS ONE*.

[11] Taheri F, Kazemi T, Chahkandi T, Namakin K, Zardast M, Bijari B (2013). Prevalence of overweight, obesity and central obesity among elementary school children in Birjand, East of Iran, 2012. *Journal of Research in Health Sciences*.

[12] Farrokh-Eslamlou HR (2013). Geographical distribution of nutrition deficiency among children under five years old in the west Azerbaijan province. *Iran Urmia Medicine Journal*.

[13] Veghari G (2012). The relationship of ethnicity, socio-economic factors and malnutrition in primary school children in North of Iran: a cross-sectional study. *Journal of Research in Health Sciences*.

[14] Esfarjani F, Roustaee R, Mohammadi-Nasrabadi F, Esmaillzadeh A (2013). Major dietary patterns in relation to stunting among children in Tehran, Iran. *Journal of Health, Population and Nutrition*.

[15] Santaliestra-Pasías AM, Rey-López JP, Moreno Aznar LA (2013). Obesity and sedentarism in children and adolescents: what should be bone?. *Nutricion Hospitalaria*.

